# Efficacy and Safety of Berberine Alone for Several Metabolic Disorders: A Systematic Review and Meta-Analysis of Randomized Clinical Trials

**DOI:** 10.3389/fphar.2021.653887

**Published:** 2021-04-26

**Authors:** Yu Ye, Xiufen Liu, Ninghua Wu, Yanqi Han, Jiawen Wang, Yuandong Yu, Qingjie Chen

**Affiliations:** ^1^ Hubei Key Laboratory of Diabetes and Angiopathy, Hubei University of Science and Technology, Xianning, China; ^2^ Basic Medical College, Hubei University of Science and Technology, Xianning, China; ^3^ Department of Oncology, Renmin Hospital, Hubei University of Medicine, Shiyan, China

**Keywords:** meta-analysis, systematic review, metabolic diseases, randomized clinical trials, berberine

## Abstract

**Background:** Metabolic activity is the basic life activity of organisms and the fundamental for maintaining body functions. With the improvement of living standards, the incidence of metabolic disorder is also increasing. At present, most of the clinical treatment strategies and meta-analysis for metabolic disorder uncover that combined medicines with berberine ameliorate several metabolic disorders. However, evidence to disclose the therapeutic effect of berberine treatment alone and the possible factors affecting the efficacy is limited. Therefore, we have formulated strict inclusion criteria and selected more reliable data for meta-analysis through more refined screening strategies to provide evidence and guidance for clinical decision-making and understand the effect of berberine treatment alone and the factors affecting its efficacy.

**Methods and results:** Using meta-analysis of “Cochrane Handbook for Systematic Reviews of Interventions” as guidelines, we searched PubMed, GeenMedical, Cochrane library, and china national knowledge infrastructure (CNKI) for trials reporting clinical treatment data of berberine. Another 417 trials were included through other sources to increase confidence in results. Among the 1,660 related documents retrieved from the four databases, 18 eligible documents were selected for analysis. Given the differences in trial design and measurement units, we used the standardized mean difference (SMD) method to eliminate the differences and then summarize the data for analysis. The main factors are triglyceride (TG), total cholesterol (TC), low-density lipoprotein (LDL), high-density lipoprotein (HDL), homeostasis model assessment-insulin resistance (HOMA-IR), and fasting plasma glucose (FPG). Random-effect model analysis was performed: TG (SMD: 0.94; 95%CI: 0.49,1.38; *p* = 0.00), TC (SMD: 1.06; 95%CI: 0.64, 1.48; *p* = 0.00), LDL (SMD: 1.77; 95%CI: 1.11,2.44; *p* = 0.00), HDL (SMD: −1.59; 95%CI: −2.32, −0.85; *p* = 0.00), HOMA-IR (SMD: 1.25; 95%CI: 0.25,2.24; *p* = 0.01), and FPG (SMD: 0.65; 95%CI: 0.28,1.03; *p* = 0.00). This study aimed to conduct a systematic review and meta-analysis of the literature to evaluate the therapeutic effect of berberine singly on metabolic diseases.

**Conclusion:** Berberine can improve obesity and hyperlipidemia by reducing TG, TC, and LDL and increasing HDL; reduce insulin resistance to improve type Ⅱ diabetes; and prevent diabetic encephalopathy.

## Introduction

The metabolic disorder discussed in this review primarily includes nonalcoholic fatty liver disease (NAFLD), type 2 diabetes, impaired glucose tolerance (prediabetes), polycystic ovarian syndrome (PCOS), and hyperlipidemia. Previous studies have demonstrated that metabolic disorders are prone to diabetic encephalopathy and atherosclerosis (Barenbrock et al., 1995), which will generate Alzheimer’s disease and coronary heart disease (Razay et al., 2007). NAFLD is closely related to type 2 diabetes and dyslipidemia (Marchesini and Babini, 2006). Characteristic changes in patients with metabolic disorders include a decrease in serum high-density lipoprotein (HDL) or an increase in serum total cholesterol (TC), triglyceride (TG), low-density lipoprotein (LDL), fasting plasma glucose (FPG), and homeostasis model assessment-insulin resistance (HOMA-IR).

At present, statins are the widely used lipid-lowering drugs worldwide. Nonetheless, its side effects can cause high blood glucose; thus, it cannot be used alone for hyperlipidemia with diabetes (Sukhija et al., 2009), and a common clinical side effect is memory and cognitive impairment. The effect refers to some unusual swelling in the neurons of patients who take statins, leading to the occurrence of memory and cognitive impairment. This effect has become an independent risk factor for induced cognitive impairment (Liu et al., 2015). At this stage, the common clinical treatment strategy is mostly the combined use of hypolipidemic and hypoglycemic drugs. However, the combined use of such drugs can cause greater risk of side effects compared with the single use of drug alone. The combination of these two drugs may increase the risks of occurrence of cognitive impairment (Suraweera et al., 2016). Therefore, we will summarize various databases on the therapeutic effect of berberine alone on metabolic disorders and conduct a systematic meta-analysis to provide a clinical medication guide and comprehensively understand the therapeutic effect of berberine alone and the factors affecting its therapeutic effect.

Berberine is an isoquinoline alkaloid compound extracted from the traditional Chinese medicine *Coptis chinensis*, and modern research has proven that it has multiple pharmacological activities (Li et al., 2018; Neag et al., 2018; Belwal et al., 2020). Recently, basic research has proven that berberine can be used to lower the blood glucose level (Liang et al., 2019), improve insulin resistance (Lou et al., 2011), improve hyperlipidemia (Li et al., 2016), and prevent mild cognitive impairment (Kumar et al., 2016). This feature improves the shortcomings of the combination of statins and metformin and shows potential as a new first-line treatment drug. This article primarily monitors the changes of HDL, TC, TG, LDL, FPG, and HOMA-IR data of patients to make clinical decision analysis on the effect of berberine, thereby providing a guiding evaluation for clinical treatment.

## Materials and Methods

### Data Source

**FIGURE 1:**
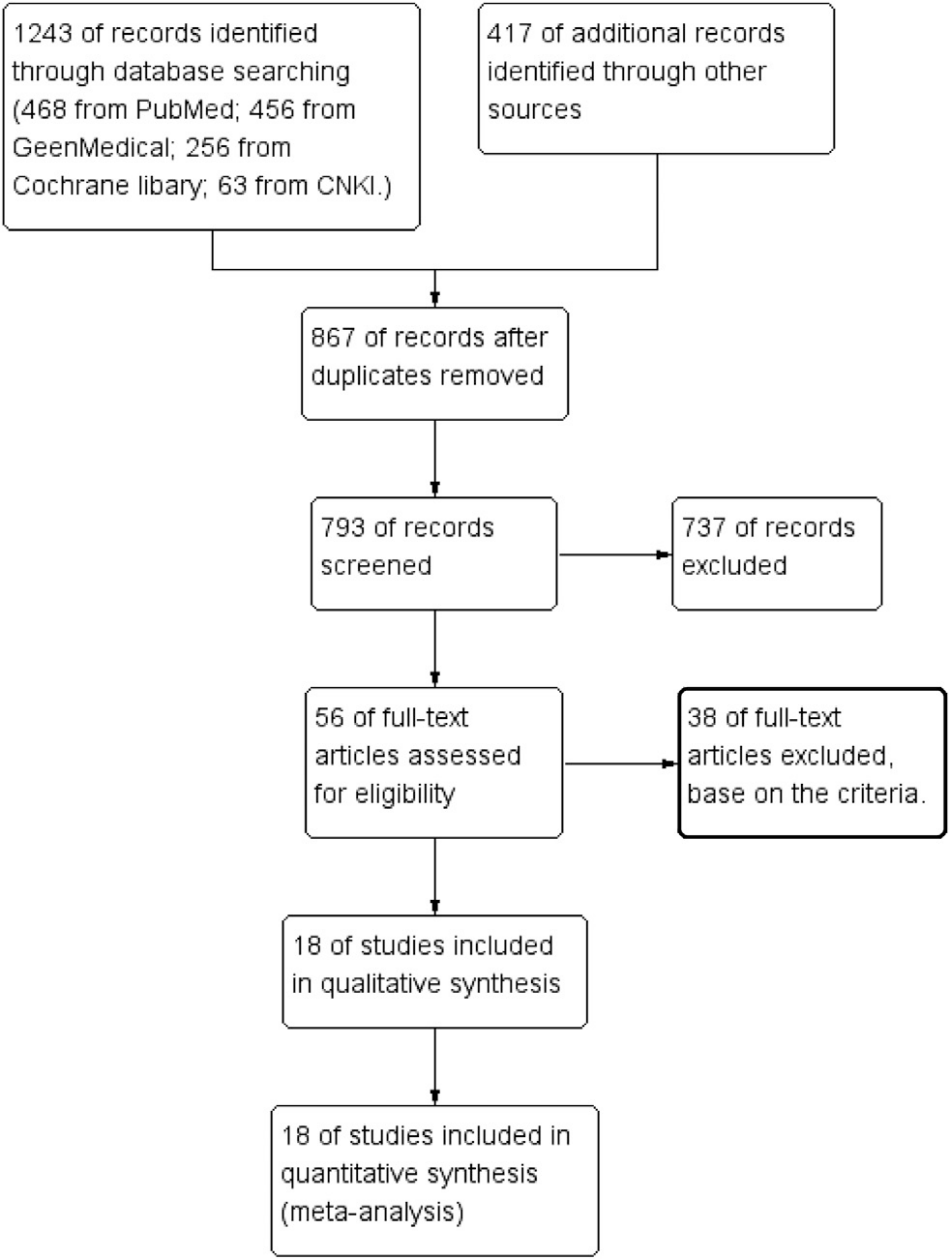
Flow diagram.

We used a combination of medical subject headings (Mesh) terminology and keywords to search four databases, namely, PubMed, Cochrane, GeenMedical, and China National Knowledge Infrastructure (CNKI), for the original studies about berberine treatment of type 2 diabetes, hyperlipidemia, and other metabolic disorders for randomized controlled trials (RCTs) without any limitations of time (1927–2021) or language. Meta-analysis was in accordance with the Cochrane Handbook for Systematic Reviews of Interventions. Another 417 additional records were included through other sources to fully incorporate all data (Figure 1).

### Search Strategy

The Search strategy is (Berberine OR Berberine Alkaloids OR Berberine hydrochloride OR Berberidaceae OR Berberis vulgaris L. OR Coptidis Rhizoma OR Berberis) AND (diabetes OR metabolic syndrome OR hyperinsulinemia OR hyperglycemia OR diabetes mellitus OR insulin resistance OR glycemic OR glycaemic OR hyperlipidemia OR hypertension OR hypercholesterolemia OR hypertriglyceridemia OR lipid metabolism OR dyslipidemia OR overweight OR obese OR obesity OR hepatic adipose infiltration OR low-density lipoprotein cholesterol OR low-density lipoprotein cholesterol OR high-density lipoprotein cholesterol OR LDL-c OR HDL-c OR total cholesterol OR TC OR triglycerides OR TC OR adipokine OR adiponectin OR adiponectins OR leptin OR leptins) AND (human [MeSH Terms] OR Clinical Trial OR Controlled Clinical Trial). A total of 468 articles were retrieved using PubMed, 456 articles were detected by GeenMedical, 256 articles were detected by Cochrane library, and 63 articles were detected by CNKI.

### Data Extraction and Study Outcomes

Our inclusion criteria were as follows: 1) study design must be a RCT of placebo controlled or parallel controlled; 2) the patients must have a metabolic disorder; 3) lifestyle intervention must be the same in the control group and trial group; 4) data of monitoring patients before and after HDL\TC\TG\LDL\FPG\HOMA-IR treatment; 5) the drug in the trial group must be berberine without any combination; 6) the drug dose in the trial group must be greater than the minimum effective dose. The exclusion criteria were as follows: 1) repeated papers in each database, 2) combined use of drugs, 3) non-clinical trial papers, 4) literature without data, 5) different lifestyle interventions used in the control and trial groups, and 6) notable deviation of research data with low credibility.

### Data Quality Assessment

According to the Cochrane Handbook for Systematic Reviews of Interventions, assessments of the quality of the selected studies involved six facets: random sequence generation, allocation concealment, blinding of participants and personnel, blinding of outcome assessment, incomplete outcome data, and selective reporting (Higgins et al., 2011; Yuan et al., 2019).

### Data Synthesis and Analysis

The screening and extraction of trial data were independently and simultaneously performed by Yu Ye and Xiufen Liu. The differences in data extraction were resolved through consultation between the two investigators. In general, the baseline and after-treatment data were compared; however, the study design of Wang Li (Wang et al., 2016) and Giuseppe Derosa (Derosa et al., 2013) had a run-in period and washout period before the treatment to 1) eliminate previous treatment drugs that may affect the treatment effect, 2) screen qualified patients to participate in the trial, 3) eliminate the effects of the first phase of the drug, and then perform the second phase of treatment. For these two sets of data, we used wash-out period data as a baseline comparison with after-treatment data. We extracted mean, SD, and sample size (N) from the literature and calculated the mean difference (mean_treatment_ − mean_baseline_), SD difference (square root (SD_treatment_)^2^ + (SD_baseline_)^2^ − 2R × SD_treatment_ × SD_baseline_, presuming a correlation coefficient R = 0.5), and TOTAL = N_control_ + N_trial_ (Yuan et al., 2019). Given the difference in trial design and measurement unit, the data adopted the standard mean difference (SMD) to eliminate the difference (Riley et al., 2011). We used RevMan5.2 software, random-effect models, or fixed-effect models (selection criteria is heterogeneity test I^2^ > 75% for high heterogeneity goes random-effect models, I^2^ < 25% for low heterogeneity goes fixed-effect models) to calculate the SMD and 95%CI. *p* < 0.05 is considered statistically significant, and a forest graph was used to analyze the effect size.

### Subgroup Analysis

We conducted the following subgroup analyses to ascertain the causes of heterogeneity and assess the factors that may affect the effectiveness of treatment: 1) treatment period, 2) treatment dose, and 3) study design (placebo/parallel). We grouped all data of TG, TC, LDL, HDL, HOMA-IR, and FPG according to the abovementioned three procedures and then analyzed the data in each subgroup to obtain the following table (Supplementary Table S1). Among the factors, TG, TC, LDL, HDL, HOMA-IR, and FPG were random-effect models (I^2^ ≥ 75%).

## Results

### Search Result

Screening was performed by passing 1,660 papers through title and abstract review; precluding duplicate papers, non-clinical trial papers, and irrelevant papers; selecting 56 papers for full-text review; and ultimately excluding articles with non-data, drug combination treatment, different life intervention strategy, non-blank, or placebo controlled. The remaining 16 articles met the requirements (Supplementary Table S2).

### Data Selection

In evaluating the therapeutic effect of berberine on metabolic disorders, we selected four commonly and clinically used blood lipid tests as indicators to measure the effect of berberine in lowering blood lipids. Moreover, FPG and HOMA-IR were selected as indicators to measure the therapeutic effect of berberine in lowering blood sugar and insulin resistance. After full-text review, we extracted 18 TG, 17 TC, 16 LDL, 14 HDL, 6 HOMA-IR, and 15 FPG data from the literature (Supplementary Table S3).

### Risk of Bias Assessment

**FIGURE 2:**
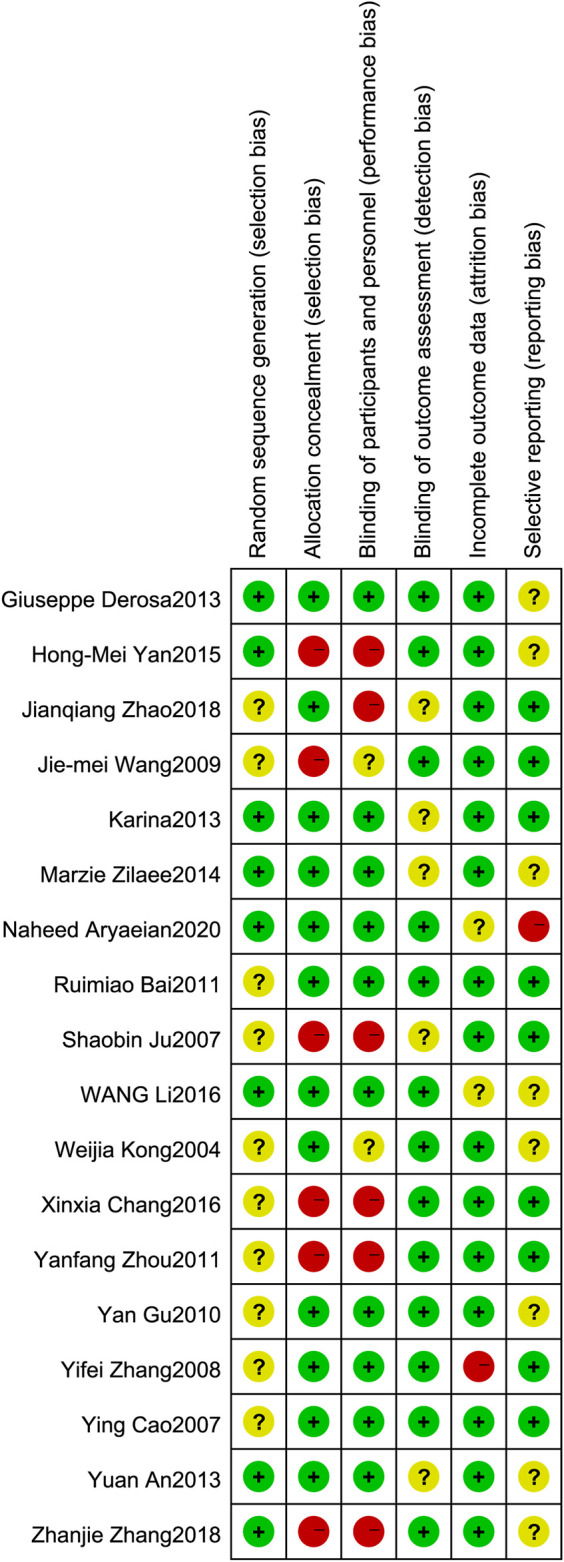
Risk of bias assessment. The details were related to the six domains that contained (1) random sequence generation (selection bias): the literature proclaims that the random sequence method is considered low risk. (2) Allocation concealment (selection bias): explain that allocation concealment or placebo-controlled experiments are considered low risk. (3) Blinding of participants and personnel (performance bias): the literature states that a double-blind study design is considered low risk. (4) Blinding of outcome assessment (detection bias): there is a return visit record or related explanation in the article that is considered low risk. (5) Incomplete outcome data (attrition bias): no outliers during the trial are considered low risk. (6) Selective reporting (reporting bias): no selective report treatment data are considered low risk.

All of the 18 included trials have proclaimed a randomized trial; however, only 8 trials proclaimed the method of randomization. Eight trials specified a double-blind study design. Of the 18 included trials, 9 were placebo controlled (lifestyle intervention and berberine vs. lifestyle intervention and placebo), and 9 were parallel controlled (lifestyle intervention and berberine vs. lifestyle intervention, Figure 2).

### Triglyceride

**FIGURE 3:**
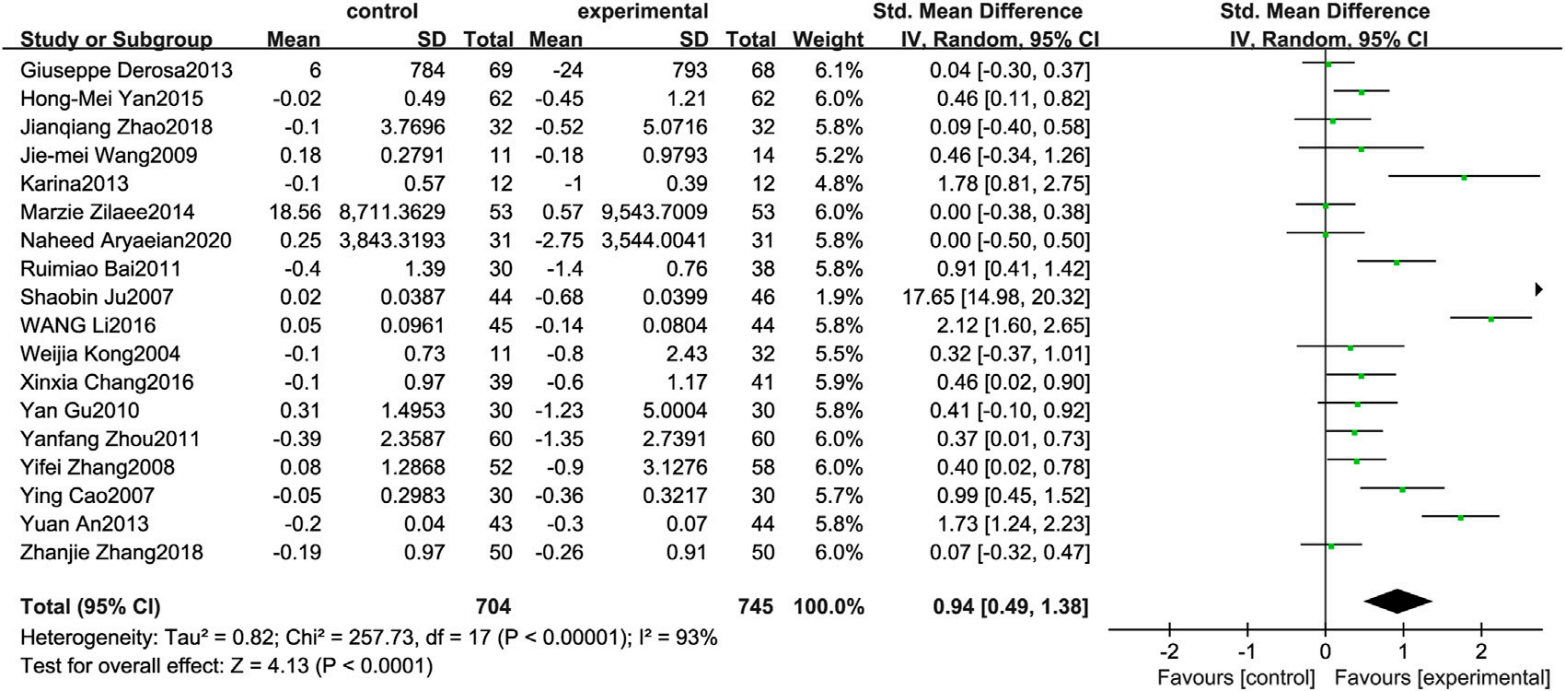
Meta-analysis and migration analysis of the effect of berberine on triglyceride. Forest plot illustrates the differences in changes in triglyceride in adults with metabolic disease who did or did not receive berberine in 15 trials (*n* = 1,231). The mean difference (blue squares), 95% CI (horizontal lines through blue squares), and pooled-effect sizes (green diamonds) were presented using random-effect Hedges models.

Figure 3 illustrates the efficacy of berberine treatment on TG. It is reported with 704 volunteers in the control group and 745 in the trial group using a randomized model. After standardization, the TG concentration of the trial group decreased by 0.94 (95%CI: 0.49, 1.38) compared with the control group. The I^2^ value was 93%, and the *p*-value was 0.00. The data indicate that berberine can reduce the TG level of the patients (Figure 3).

### Total Cholesterol

**FIGURE 4:**
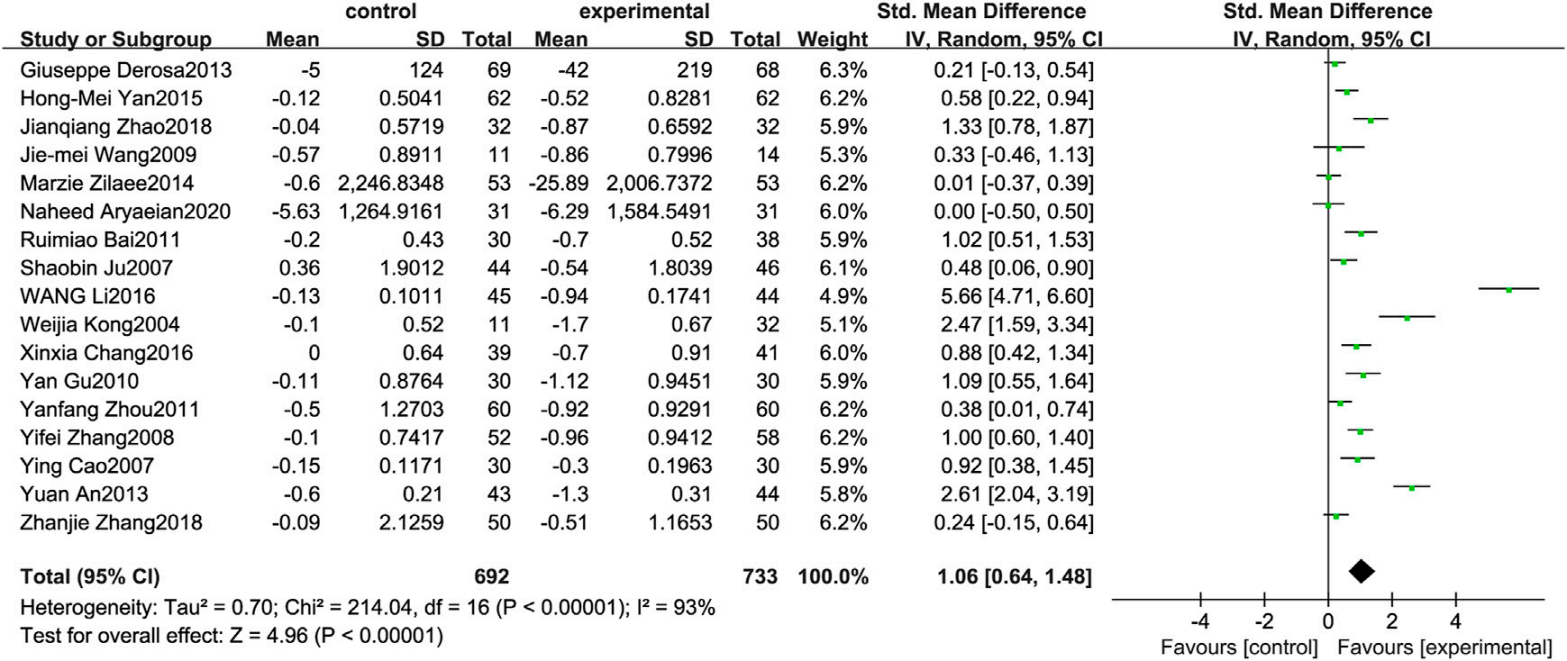
Meta-analysis and migration analysis of the effect of berberine on total cholesterol. Forest plot illustrates the differences in changes in total cholesterol in adults with metabolic disease who did or did not receive berberine in 15 trials (*n* = 1,297). The mean difference (blue squares), 95% CI (horizontal lines through blue squares), and pooled-effect sizes (green diamonds) were presented using random-effect Hedges models.

Figure 4 illustrates the efficacy of berberine treatment on TC. It is reported with 692 volunteers in the control group and 733 in the trial group using a randomized model. After standardization, the TC concentration of the trial group decreased by 1.06 (95%CI: 0.64, 1.48) compared with the control group. The I^2^ value was 93%, and the *p*-value was 0.00. The data demonstrate that berberine can reducethe TC level of the patients (Figure 4).

### Low-Density Lipoprotein

**FIGURE 5:**
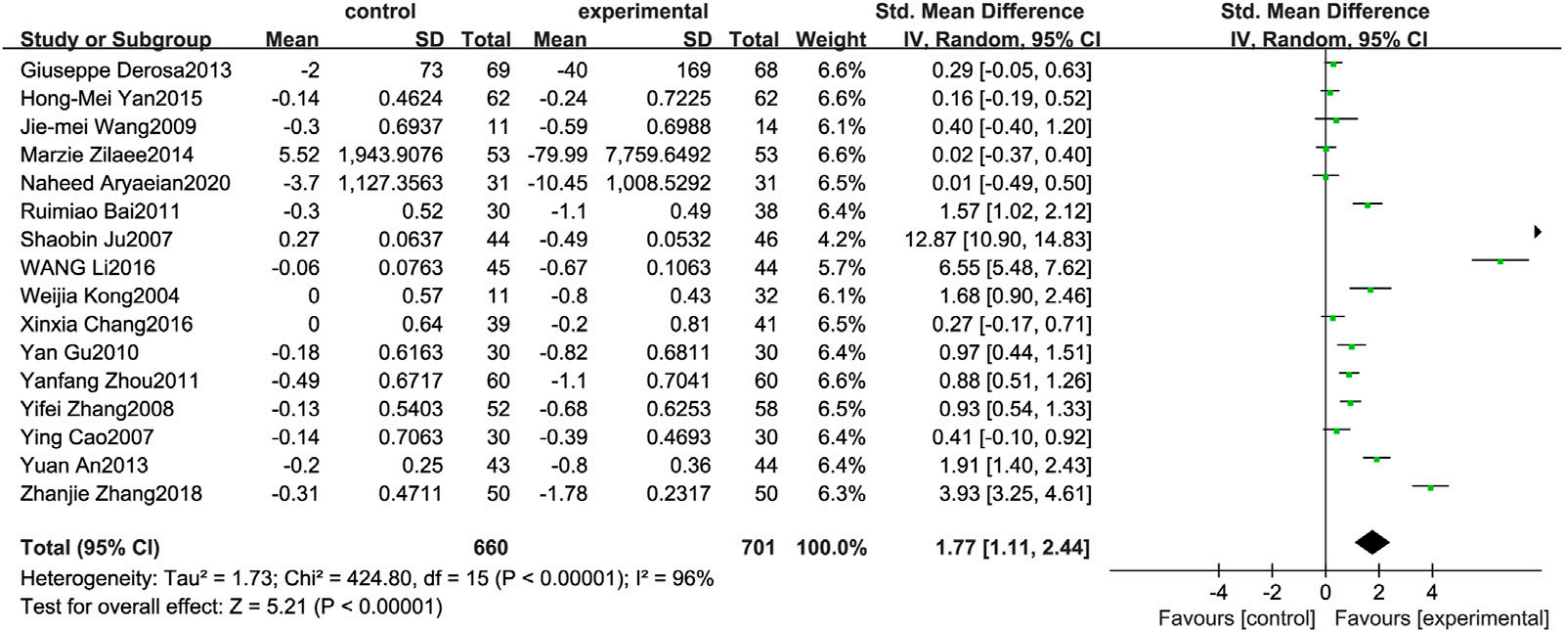
Meta-analysis and migration analysis of the effect of berberine on low-density lipoprotein. Forest plot illustrates the differences in changes in low-density lipoprotein in adults with metabolic disease who did or did not receive berberine in 14 trials (*n* = 1,233). The mean difference (blue squares), 95% CI (horizontal lines through blue squares), and pooled-effect sizes (green diamonds) were presented using random-effect Hedges models.

Figure 5 illustrates the efficacy of berberine treatment on LDL. It is reported with 660 volunteers in the control group and 701 in the trial group using a randomized model. After standardization, the LDL concentration of the trial group decreased by 1.77 (95%CI: 1.11, 2.44) compared with the control group. The I^2^ value was 96%, and the *p*-value was 0.00. The chart illustrates that berberine can reduce the LDL level of the patients (Figure 5).

### High-Density Lipoprotein

**FIGURE 6:**
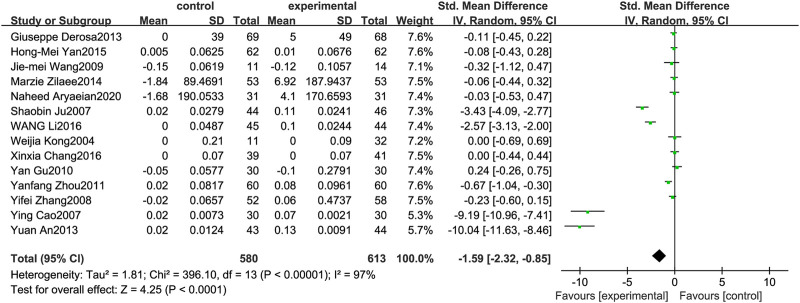
Meta-analysis and migration analysis of the effect of berberine on high-density lipoprotein. Forest plot illustrates the differences in changes in high-density lipoprotein in adults with metabolic disease who did or did not receive berberine in 13 trials (*n* = 1,133). The mean difference (blue squares), 95% CI (horizontal lines through blue squares), and pooled-effect sizes (green diamonds) were presented using random-effect Hedges models.

Figure 6 illustrates the efficacy of berberine treatment on HDL. It is reported with 580 volunteers in the control group and 613 in the trial group using a randomized model. After standardization, the HDL concentration of the trial group increased by 1.59 (95%CI: −2.32, −0.85) compared with the control group. The I^2^ value was 97%, and the *p*-value was 0.00. The chart illustrates that berberine can improve the HDL level of the patients (Figure 6).

### Homeostasis Model Assessment-Insulin Resistance

**FIGURE 7:**
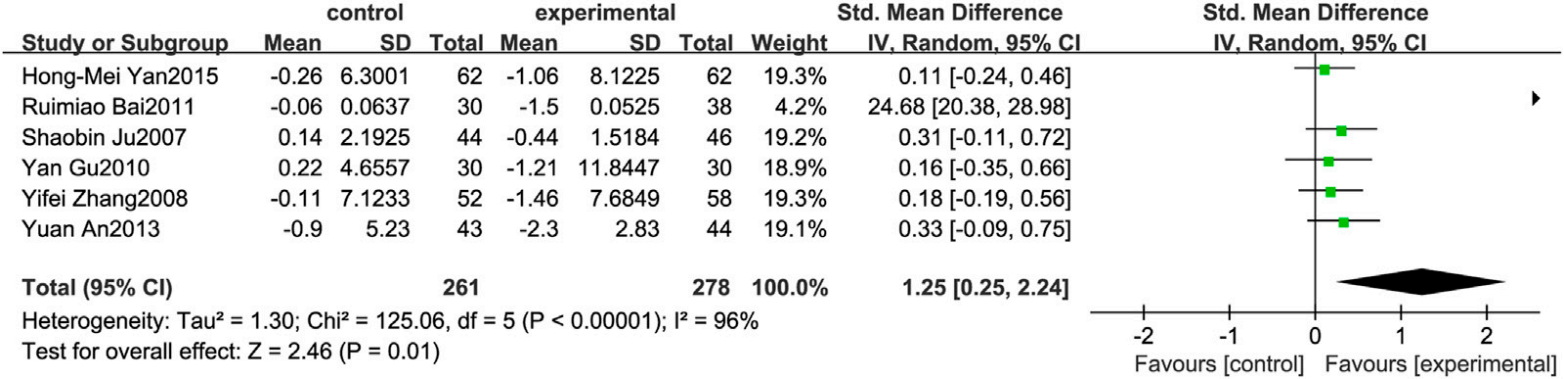
Meta-analysis and migration analysis of the effect of berberine on homeostasis model assessment of insulin resistance. Forest plot illustrates the differences in changes in homeostasis model assessment of insulin resistance in adults with metabolic disease who did or did not receive berberine in 5 trials (*n* = 471). The mean difference (blue squares), 95% CI (horizontal lines through blue squares), and pooled-effect sizes (green diamonds) were presented using fixed-effects inverse-variance models.

Figure 7 illustrates the efficacy of berberine treatment on HOMA-IR. It is reported with 261 volunteers in the control group and 278 in the trial group using a randomized model. After standardization, the HOMA-IR concentration of the trial group decreased by 1.25 (95%CI: 0.25, 2.24) compared with the control group. The I^2^ value was 96%, and the *p*-value was 0.01. The chart illustrates that berberine can reduce the HOMA-IR level of the patients (Figure 7).

### Fasting Plasma Glucose

**FIGURE 8:**
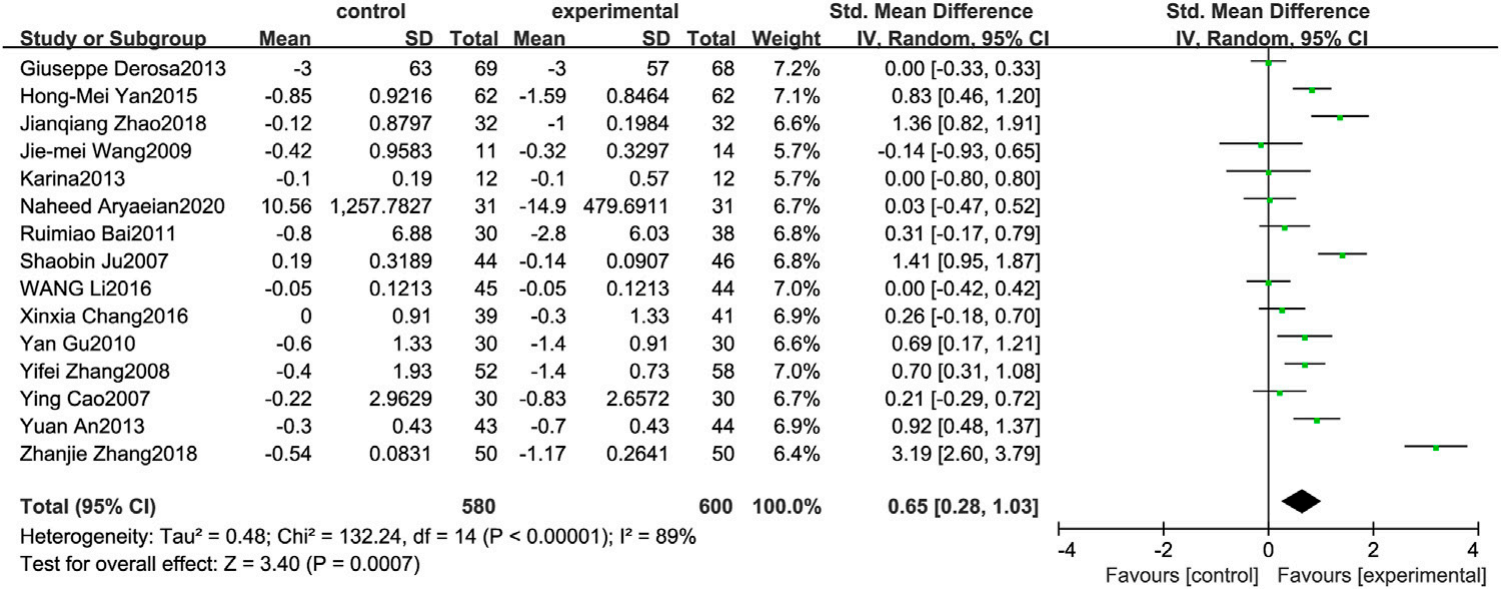
Meta-analysis and migration analysis of the effect of berberine on fasting plasma glucose. Forest plot illustrates the differences in changes in fasting plasma glucose in adults with metabolic disease who did or did not receive berberine in 13 trials (*n* = 1,052). The mean difference (blue squares), 95% CI (horizontal lines through blue squares), and pooled-effect sizes (green diamonds) were presented using random-effect Hedges models.

Figure 8 illustrates the efficacy of berberine treatment on FPG. It is reported with 580 volunteers in the control group and 600 in the trial group using a randomized model. After standardization, the FPG concentration of the trial group decreased by 0.65 (95%CI: 0.28, 1.03) compared with the control group. The I^2^ value was 89%, and the *p*-value was 0.00. The data shows that berberine can reduce the FPG level of the patients (Figure 8).

### Subgroup Analysis Result

Drug users have different physiological conditions such as gender, age, individual differences, and dietary structure, and the effects of taking the same drug are different. In addition, the treatment period and treatment dose will affect the drug treatment effect. Our subgroup analysis explored the possible reasons based on three aspects: treatment period, treatment dose, and study design (Table 1).

In TG subgroup analysis, overall differences were found in the therapeutic effects of different treatment periods (*p* = 0.02), whereas no difference was found in the therapeutic effects of different treatment doses (*p* = 0.08) and study designs (*p* = 0.20).

In TC subgroup analysis, overall differences were found in the therapeutic effects of different treatment periods (*p* = 0.01), whereas no difference was found in the therapeutic effects of different treatment doses (*p* = 0.60) and study designs (*p* = 0.06). Treatment period is the main factor causing heterogeneity.

In LDL subgroup analysis, differences were observed in the therapeutic effects of different treatment periods (*p* = 0.04) and treatment doses (*p* = 0.00), whereas no difference was observed in the therapeutic effects of different study designs (*p* = 0.30).

In HDL subgroup analysis, no difference was observed in the treatment effect of different treatment periods (*p* = 0.25), treatment doses (*p* = 0.08), and study designs (*p* = 0.42).

HOMA-IR subgroup analysis showed a notable difference in heterogeneity through different treatment periods, treatment doses, and study designs, revealing that more data are needed to enrich credibility.

In FPG subgroup analysis, no difference was observed in the treatment effects of different treatment periods (*p* = 0.07), treatment doses (*p* = 0.28), and study designs (*p* = 0.11).

## Discussion

Berberine can be used as an alternative treatment for patients who do not tolerate statins because of its lipid-lowering effects (Banach et al., 2018). The findings of the present meta-analysis demonstrated that berberine alone can reduce TG, TC, LDL, HDL, FPG, and HOMA-IR levels in patients with metabolic disorders, and this effect was observed in healthy participants. This meta-analysis on the efficacy and safety of berberine for several metabolic disorders leads to the propel guidelines of berberine in clinical practice. These effects indicate that berberine has the potential to treat metabolic disorders such as type 2 diabetes complicated with hyperlipidemia. Compared with traditional statins and metformin, berberine also has great benefits in improving mild cognitive impairment, effectively preventing secondary or the occurrence of drug-induced metabolic encephalopathy. In the abovementioned clinical treatment process, only a few patients have occasional abdominal pain, and drug safety is significantly higher than the original treatment measures. Compared with previous studies, we discuss the therapeutic effect of berberine alone in the treatment of metabolic disorders for the first time. Three subgroup analyses were conducted on the basis of the treatment period, treatment dose, and study design (placebo/parallel) to reveal the factors affecting the therapeutic effect. Treatment period and dose are the main factors causing heterogeneity, and study design has almost no effect on heterogeneity. Extending the berberine treatment period (>3 months) will significantly increase the therapeutic effect. Based on the effects of dose and time on different monitoring indicators, subgroup analysis can also be used as a clinical treatment guide. TC is a risk factor for patients with hyperlipidemia, and atherosclerosis dominated by elevated TC is a key factor affecting the treatment effect. The results show that the treatment period is a key factor affecting the treatment effect in patients with metabolic disorders and coronary heart disease dominated by elevated TG. In addition, elevated LDL can affect atherosclerosis formation and cause coronary heart disease. Therefore, as a risk factor for atherosclerosis, LDL can be used to assess the risk of coronary heart disease. Our data indicate that the treatment period and dose for patients with elevated LDL are key factors affecting the therapeutic effect. At present, no sufficient evidence is found to prove that treatment time and treatment dose affect the treatment effect in patients with insulin resistance and/or abnormal blood glucose levels.

Berberine is clinically safe and well-tolerated by the human body. Few adverse reactions are reported, and no negative effect is observed on participants’ diet. Research by Yun S. Lee et al. (Lee et al., 2006) showed that berberine can inhibit fat-forming and lipogenic genes of fat, thereby reducing fat production. By increasing the expression of uncoupling protein mRNA in skeletal muscles, heat production and oxygen consumption are increased, and glucose and fat metabolism is accelerated. Berberine is also an AMPK agonist, and it increases energy production and reduces energy storage by activating AMPK. The activation of AMPK can normalize the imbalance of lipid, glucose, and energy and improve the metabolic imbalance caused by metabolic disorders (Srivastava et al., 2012). Activating AMPK can also promote GLUT4 translocation, indirectly speeding up the transport of glucose in the serum (Burcelin et al., 2003) and free fatty acids to the mitochondria by increasing ACC phosphorylation, both of which contribute to the reduction of glucose and lipids (Zhang et al., 2019). In addition, berberine-mediated AMPK activation has an anti-inflammatory effect, which improves insulin resistance. Berberine can also stabilize LDLR mRNA and prolong half-life (Lou et al., 2011; Yang et al., 2016). The stable expression of LDLR can increase the clearance rate of plasma LDL through receptor-mediated endocytosis, thereby reducing LDL (Kong et al., 2004). Our meta-analysis results correspond to the theory that berberine activates AMPK to regulate metabolism. Berberine activates AMPK to reduce fat production and body fat ratio, improves insulin sensitivity, and promotes glucose transport. Collectively, these effects cause the fat and sugar in patients with metabolic disorders to change from accumulation to decomposition. Furthermore, Asbaghi et al. found that weight loss and anti-inflammatory effects related to berberine intake may play an indirect role in improving the clinical symptoms of metabolic disorders (Asbaghi et al., 2020).

Berberine alone in patients with metabolic disorders can play a good therapeutic effect containing blood lipid-lowering, blood sugar-lowering, and insulin resistance amelioration, and the therapeutic effect becomes conspicuous with treatment time > 3 months. No clear direct evidence is found to prove the effects of age and the severity of the disease on drug efficacy, but evidence supports the use of berberine therapy in older people with metabolic disorders.

Our study still has the following limitations: 1) random errors may be found in the inclusion of the sample; 2) differences are observed in the characteristics of RCT samples, such as gender and age differences; 3) metabolic disorders are often combined with several diseases. Subgroup analysis can be performed in the future to evaluate the therapeutic effect of berberine on a single disease. 4) Life interventions, including diet intervention and exercise intervention of each study design, are not exactly the same. This study does not exclude the possibility that life intervention can improve monitoring indicators. 5) Some articles do not indicate whether the research object is newly diagnosed or has been sick in the past. The frequency of return visits and specific procedures during the experiment are not mentioned. These factors may also lead to the appearance of bias. 6) Differences may be found in the efficacy and metabolism of drugs for different races, and more data are needed to verify the results. 7) The current study is not registered, and it may have a small deviation, but we still strictly follow the steps of a systematic review.

## Data Availability

The original contributions presented in the study are included in the article/Supplementary Material, further inquiries can be directed to the corresponding authors.
